# Real-world pharmacovigilance analysis of the association between multiple sclerosis disease-modifying therapies and intervertebral disc herniation

**DOI:** 10.3389/fphar.2026.1877458

**Published:** 2026-06-22

**Authors:** Nan Yang, Hui Liu, Yue Zhou, Haoyu Feng

**Affiliations:** 1 Shanxi Bethune Hospital, Shanxi Academy of Medical Sciences, Tongji Shanxi Hospital, Third Hospital of Shanxi Medical University, Taiyuan, China; 2 Hospital of China Railway 12th Bureau Group, Taiyuan, China; 3 Xinqiao Hospital ARMY Medical University, Chongqing, China

**Keywords:** FAERS database, intervertebral disc herniation, natalizumab, ocrelizumab, pharmacovigilance analysis, teriflunomide

## Abstract

**Background:**

This study aims to investigate the potential association between 13 disease-modifying immunosuppressive drugs used to treat multiple sclerosis and intervertebral disc herniation, thereby filling a gap in pharmacovigilance research in this field.

**Materials and methods:**

We extracted adverse event reports from the FAERS database covering 2005 to 2025. Disproportionality analyses using four established algorithms (ROR, PRR, EBGM and IC) with unified predefined thresholds were performed. We also conducted subgroup analyses and explored the temporal characteristics of adverse event onset. Results: Teriflunomide, natalizumab and ocrelizumab have shown positive disproportionality signals for intervertebral disc herniation. And these signals merely reflect statistical associations and do not confirm causality. Among these, men and young-to-middle-aged adults populations presented relatively higher disproportionality signal values. Furthermore, teriflunomide-associated intervertebral disc herniation exhibits characteristics of early-onset risk, whereas there is no clear temporal clustering of events associated with natalizumab and ocrelizumab.

**Conclusion:**

Three medications demonstrated positive disproportionality reporting signals for intervertebral disc herniation, which require further validation. Clinicians are suggested to raise vigilance on spinal adverse events and tailor therapeutic strategies when prescribing these agents for multiple sclerosis management.

## 1 Introduction

Intervertebral disc herniation is one of the most common degenerative conditions in the field of spinal surgery. With a persistently high incidence, this condition now affects more young people and has emerged as a major public health concern (Benzakour and Benzakour, 2019; Yang et al., 2025b). The core pathological changes of this condition involve disc degeneration, annular rupture, and protrusion of the nucleus pulposus compressing the nerve roots or cauda equina. Clinical manifestations include persistent pain and radiating numbness; in severe cases, it can lead to urinary and bowel dysfunction, saddle area sensory abnormalities, or even paraplegia, significantly reducing patients’ quality of life (Wanderman et al., 2020; Chu et al., 2023). The pathogenesis of intervertebral disc herniation is multifactorial. Currently, the academic community generally recognizes age-related degenerative changes in the intervertebral discs as a causative factor. However, with the progress of clinical research, accumulating data indicate that environmental factors are closely correlated with the onset and progression of intervertebral disc herniation (Jiang et al., 2025; Yang et al., 2025a). Long-term use of certain medications may also be associated with this condition, an association that requires further exploration.

Multiple sclerosis (MS) is a chronic autoimmune disease characterized by multifocal demyelination of the central nervous system. The disease typically affects young-to-middle-aged adults, significantly impacting patients’ neurological function and long-term prognosis (Files et al., 2015; Howard et al., 2016; Reich et al., 2018). Currently, disease-modifying therapies (DMTs) are the primary first-line treatment for MS. A wide variety of immunosuppressive DMTs are available on the market, which can be classified into 13 types across 7 major categories based on their pharmacological mechanisms of action (Balshi et al., 2025). Due to significant variations in disease severity, disease progression, comorbidities, and individual tolerance among MS patients, there is a lack of uniform standards for clinical drug selection, resulting in a marked diversity of treatment regimens. Clinical evidence has demonstrated that disease-modifying therapies for multiple sclerosis can effectively regulate disease activity, yet they are also associated with a broad spectrum of adverse events across the immune, gastrointestinal and musculoskeletal systems. Intervertebral disc herniation is a disabling musculoskeletal disorder whose clinical manifestations overlap with those of multiple sclerosis. This condition predominantly affects young-to-middle-aged adults, a demographic that largely coincides with the typical MS patient population. Such population overlap may cause patients receiving long-term DMT treatment to overlook the onset of intervertebral disc herniation. Nevertheless, potential links between MS disease-modifying therapies and intervertebral disc herniation have not been fully elucidated, and dedicated pharmacovigilance investigations on this topic remain markedly insufficient (Caldito et al., 2021; Shirani et al., 2024).

The U.S. Food and Drug Administration Adverse Event Reporting System (FAERS) is the world’s largest and most comprehensive postmarketing pharmacovigilance database, established and maintained by the U.S. FDA. Its core function is to systematically collect, manage, and analyze adverse event reports related to drugs and therapeutic biological products submitted by healthcare professionals, patients, and pharmaceutical manufacturers worldwide (Yang et al., 2025c). Given the current state of research and existing gaps, this study aims to use the FDA Adverse Event Reporting System as a data source to systematically identify and analyze pharmacovigilance signals linking 13 disease-modifying therapies for multiple sclerosis to intervertebral disc herniation. We aimed to compare signal differences across different medications. This work intends to fill the research gap concerning their statistical correlation. It also provides evidence to support personalized medication use for MS patients, as well as the monitoring and prevention of adverse events related to intervertebral disc herniation.

## 2 Materials and methods

### 2.1 Data sources

**FIGURE 1:**
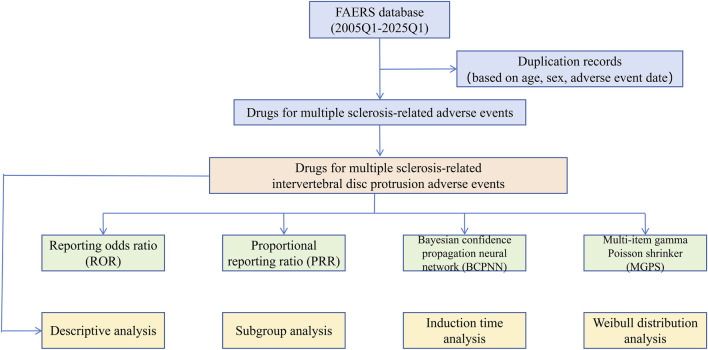
Flow chart showing the analysis process of the study.

The data for this study were sourced from the FAERS database. The study included all adverse event reports submitted to the database between 2005 and 2025 that were associated with 13 types of MS DMTs (Figure 1). The FAERS database contains core data such as patient demographic information, medication usage information, adverse event information, and reporting sources. All data have undergone standardization by the FDA to ensure data integrity and consistency. This study strictly adhered to the FAERS database usage guidelines. Duplicate reports, logically inconsistent records and entries with severe data gaps were removed. Severe data gaps were defined as the absence of key information required for disproportionality analysis, such as demographic data, medication details and verified adverse event diagnoses. Eligible reports were included in subsequent analyses. In addition to removing duplicate case reports, we also excluded records with obvious chronological inconsistencies. Specifically, we eliminated entries where the recorded end date preceded the start date, including cases where the documented drug discontinuation time was earlier than the initiation time. Additionally, in accordance with official FDA guidelines, this study utilized CASEID, PRIMARYID, and drug_seq as unique identifiers for data linkage and sorting. Furthermore, in accordance with FDA regulations, for reports with matching PRIMARYIDs, the latest FDA_DT was used as the core temporal identifier for filtering. For duplicate reports with identical FDA_DT and CASEID values, the entry with the higher PRIMARYID value was retained.

### 2.2 Identification of investigational drugs

The 13 MS DMTs included in this study are all immunosuppressive disease-modifying therapies currently in widespread clinical use. They are classified into seven major categories based on their pharmacological mechanisms of action. Specifically, these include anti-CD20 monoclonal antibodies such as ocrelizumab, ofatumumab, and rituximab; integrin inhibitors such as natalizumab; anti-CD52 monoclonal antibodies such as alemtuzumab; fumaric acid derivatives such as dimethyl fumarate and diroximel fumarate; and S1P receptor modulators such as fingolimod, siponimod, ponesimod and ozanimod; and immunosuppressive cytotoxic agents such as mitoxantrone and cladribine; and pyrimidine synthesis inhibitors such as teriflunomide. Additionally, non-immunosuppressive drugs were excluded because they may be used for symptomatic treatment of intervertebral disc herniation; to avoid confounding bias and ensure the specificity of the study data. All drugs were retrieved using MeSH subject headings to ensure the accuracy and comprehensiveness of the search results.

### 2.3 Definition and retrieval of adverse events

MedDRA version 26.1 provides a tiered medical terminology framework consisting of preferred terms (PT), high-level terms (HLT), high-level group terms (HLGT) and system organ classes (SOC). Per MedDRA adverse event coding specifications, four drug attribution categories are defined, namely PS (primary suspicion), SS (secondary suspicion), C (concomitant effect), and I (interaction). To secure reliable analytical outcomes, only cases tagged as primary suspected adverse reactions were enrolled in subsequent assessment. The targeted adverse event of this study is intervertebral disc herniation, with the corresponding retrieval term INTERVERTEBRAL DISC PROTRUSION adopted in the FAERS database. Related manifestations including disc bulging and disc prolapse were not analyzed separately, as no distinct coding differentiation exists for these conditions within the database.

### 2.4 Data processing and statistical analysis

This study first conducted a descriptive statistical analysis of the included adverse drug reaction reports to elucidate the overall distribution characteristics of adverse events. Building on this, an overall analysis of seven major drug categories was performed to identify core drug categories exhibiting potential statistical signals. Subsequently, independent pharmacovigilance analyses were conducted for each of the 13 target drugs, progressively mining and validating risk signals from the broad drug category down to individual drugs. Commonly used signal mining algorithms in pharmacovigilance-including the Reporting Odds Ratio (ROR), Proportional Reporting Ratio (PRR), Empirical Bayesian Geometric Mean (EBGM), and Information Component (IC)-were employed to systematically analyze pharmacovigilance association signals between MS DMTs and intervertebral disc herniation. Disproportionality analysis was performed using a classic 2 × 2 contingency table, where cell a represents the number of reported cases exposed to the target MS treatment drugs who developed intervertebral disc herniation, cell b represents the number of reported cases exposed to the target MS treatment drugs who did not develop intervertebral disc herniation, cell c represents the number of reported cases exposed to other drugs who developed intervertebral disc herniation, and cell d represents the number of reported cases exposed to other drugs who did not develop intervertebral disc herniation. The detailed calculation algorithms and positive threshold criteria for the four disproportionality analyses are presented in Supplementary Table S1 (Luo et al., 2025; Zhang et al., 2025). To reduce the risk of false-positive results, an association was considered a statistically significant safety signal only when all four algorithms met the positive determination criteria. Among the various algorithms, the ROR method demonstrated superior performance in bias control; therefore, it was adopted as the primary basis for this signal evaluation (Rothman et al., 2004). Additionally, the Bonferroni correction method was applied to validate the multiple comparison process, effectively controlling the probability of Type I errors. Specifically, this adjustment was performed based on the total number of PTs within each demographic subgroup for multiple testing. Furthermore, this study conducted subgroup analyses by gender (male, female). Additionally, based on the age range in which the disease is most prevalent, age stratification (≤18 years, 18–45 years, 45–65 years, >65 years) was also used for subgroup analysis to explore differences in the association characteristics between MS DMTs and intervertebral disc herniation across different populations. Furthermore, this study employed Weibull distribution analysis to examine the temporal patterns of reported intervertebral disc herniation events, thereby further elucidating the association between the two from a temporal perspective. The shape parameter and scale parameter were estimated using the fitdistrplus package (version 1.2–6) in the R statistical environment. All data processing and statistical analyses were performed using R software (version 4.4.2).

## 3 Results

### 3.1 Basic information included in the report

**FIGURE 2:**
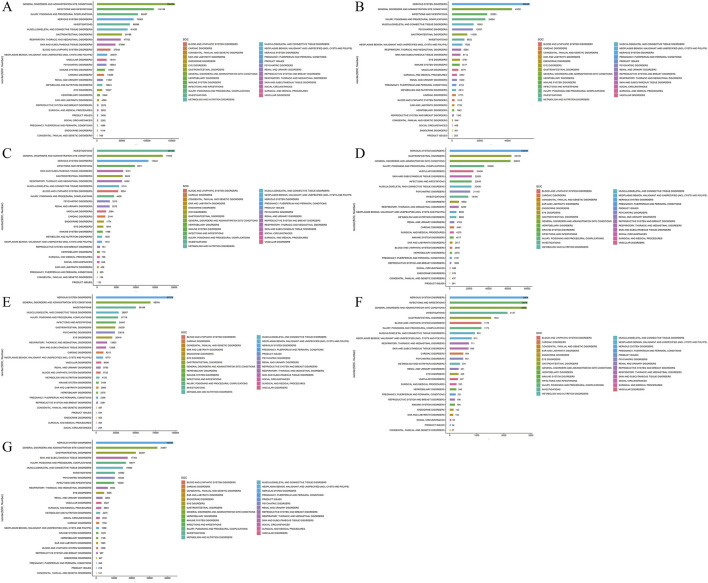
Bar chart showing the MS DMTs with positive significance at the system organ classification (SOC) level. **(A)** Anti-CD20 monoclonal antibodies; **(B)** Integrin inhibitors; **(C)** Anti-CD52 monoclonal antibodies; **(D)** Fumaric acid derivatives; **(E)** S1P receptor modulators; **(F)** Immunosuppressive cytotoxic agents; **(G)** Pyrimidine synthesis inhibitors. The horizontal axis indicates the number of reports for each SOC, and the vertical axis represents different categories of SOC.

This study extracted adverse event reports related to 13 types of MS DMTs across 7 major categories from the FAERS database for the period from 2005 to 2025 (Table 1). After data cleaning and organization, a total of 1,629 adverse event reports related to intervertebral disc herniation were ultimately included. The top two drug categories by number of reports were fumarates (n = 350) and anti-CD20 monoclonal antibodies (n = 387). Classification according to the System Organ Class (SOC) revealed that adverse drug reactions associated with multiple sclerosis disease-modifying therapies can affect 27 organ systems (Figure 2). Among these, “MUSCULOSKELETAL AND CONNECTIVE TISSUE DISORDERS” which includes intervertebral disc herniation events, is one of the primary affected system categories for adverse drug reactions of this class.

**TABLE 1:** Clinical distribution characteristics of adverse event reports associated with MS DMTs.

Characteristics (N,%)	Anti-CD20 monoclonal antibodies	Fumaric acid derivatives	S1P receptor modulators	Immunosuppressive cytotoxic agents	Pyrimidine synthesis inhibitors	Integrin inhibitors	Anti-CD52 monoclonal antibodies
Number of events	165,504	93,253	80,313	6,384	37,114	60,687	9,730
Sex, n%							
Female	83,357 (50.4%)	67,955 (72.9%)	58,689 (73.1%)	4,322 (67.7%)	27,366 (73.7%)	45,716 (75.3%)	5,535 (56.9%)
Male	42,760 (25.8%)	18,857 (20.2%)	17,383 (21.6%)	1,489 (23.3%)	6,593 (17.8%)	12,967 (21.4%)	2,586 (26.6%)
Unknown	39,387 (23.8%)	6,441 (6.9%)	4,241 (5.3%)	573 (9.0%)	3,155 (8.5%)	2004 (3.3%)	1,609 (16.5%)
Age, n%							
<18	2049 (1.2%)	330 (0.4%)	604 (0.8%)	136 (2.1%)	41 (0.1%)	474 (0.8%)	253 (2.6%)
18–64.9	57,696 (34.9%)	35,101 (37.6%)	38,924 (48.5%)	3,215 (50.4%)	20,422 (55.0%)	26,257 (43.3%)	5,855 (60.2%)
65–85	20,002 (12.1%)	4,724 (5.1%)	2,750 (3.4%)	416 (6.5%)	3,206 (8.6%)	1945 (3.2%)	442 (4.5%)
>85	736 (0.4%)	14 (0.0%)	22 (0.0%)	1 (0.0%)	21 (0.1%)	2 (0.0%)	15 (0.2%)
Unknow	85,021 (51.4%)	53,084 (56.9%)	38,013 (47.3%)	2,616 (41.0%)	13,424 (36.2%)	32,009 (52.7%)	3,165 (32.5%)
Outcome							
CA	104 (0.1%)	87 (0.1%)	88 (0.1%)	9 (0.1%)	25 (0.1%)	157 (0.3%)	11 (0.1%)
DE	14,106 (8.5%)	2,103 (2.3%)	1,106 (1.4%)	333 (5.2%)	552 (1.5%)	2,906 (4.8%)	631 (6.5%)
DS	1,383 (0.8%)	164 (0.2%)	697 (0.9%)	53 (0.8%)	764 (2.1%)	155 (0.3%)	251 (2.6%)
HO	28,618 (17.3%)	20,618 (22.1%)	8,748 (10.9%)	1,273 (19.9%)	4,493 (12.1%)	15,465 (25.5%)	2,843 (29.2%)
LT	3,386 (2.0%)	387 (0.4%)	950 (1.2%)	120 (1.9%)	223 (0.6%)	526 (0.9%)	383 (3.9%)
OT	53,564 (32.4%)	13,420 (14.4%)	24,204 (30.1%)	2,614 (40.9%)	9,545 (25.7%)	10,510 (17.3%)	3,262 (33.5%)
RI	238 (0.1%)	52 (0.1%)	19 (0.0%)	6 (0.1%)	10 (0.0%)	4 (0.0%)	5 (0.1%)
Unknown	64,105 (38.7%)	56,422 (60.5%)	44,501 (55.4%)	1976 (31.0%)	21,502 (57.9%)	30,964 (51.0%)	2,344 (24.1%)

Abbreviations: CA: congenital anomaly; DE: death; DS: disability; HO: Hospitalization-Initial or Prolonged; LT: Life-Threatening; OT: other serious important medical event; RI: required intervention to prevent permanent.

### 3.2 Signal analysis of the association between 7 major categories of MS DMTs and intervertebral disc herniation

**FIGURE 3:**
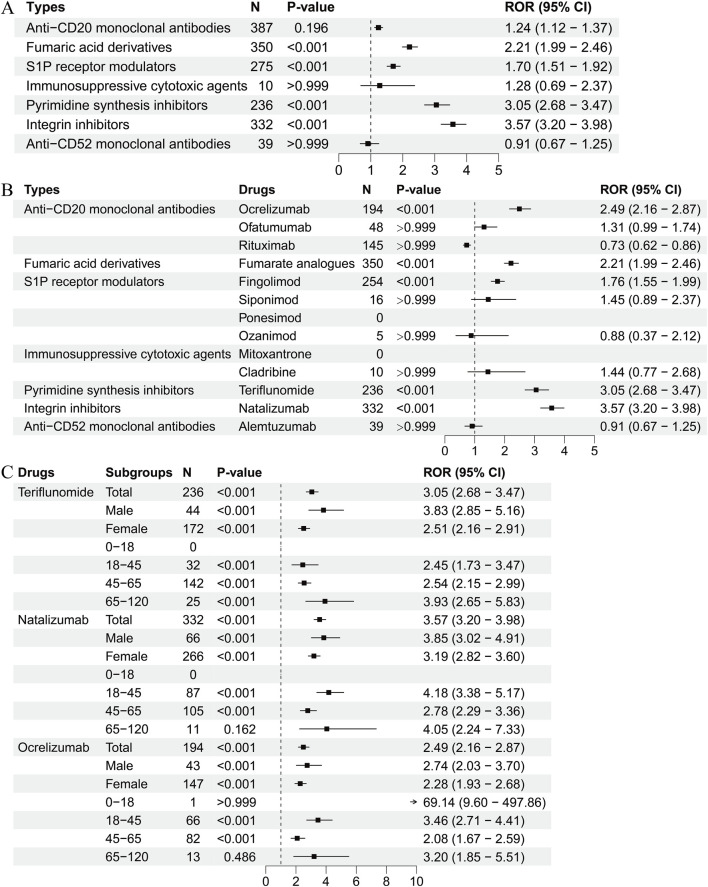
Forest plot showing the results of MS DMTs with positive significance that tested after disproportionate analysis. **(A)** 7 Major Categories of MS DMTs; **(B)** 13 Types of MS DMTs; **(C)** Subgroup analyses of 3 drugs with positive pharmacovigilance signals. The x-axis represents the magnitude of ROR signal values. ROR: Reporting Odds Ratio; 95% CI, two-sided for ROR; P value: Bonferroni-corrected P value.

The results of pharmacovigilance signal mining indicate that, among the seven major classes of MS DMTs, only the case of pyrimidine synthesis inhibitors (n = 236, ROR = 3.05, 95% CI: 2.68–3.47, P < 0.001) and integrin inhibitors (n = 332, ROR = 3.57, 95% CI: 3.20–3.98, P < 0.001) showed positive signals (Figure 3). In contrast, no positive signals related to intervertebral disc herniation were detected for the remaining five drug classes. Furthermore, disproportionality signals from spontaneous reporting systems are susceptible to reporting bias, notoriety bias, indication bias and channeling bias. Thus, caution is warranted when interpreting these results.

### 3.3 Signal analysis of the association between 13 types of MS DMTs and intervertebral disc herniation

To fully identify potential safety signals and compensate for the limitations of category-based analysis, we performed separate disproportionality analyses for all 13 individual MS DMTs. This approach improved the completeness and reliability of signal detection. Individual-drug analysis yielded significant positive signals for three agents. Besides teriflunomide (pyrimidine synthesis inhibitor) and natalizumab (integrin inhibitor), ocrelizumab (anti-CD20 monoclonal antibody) also presented a statistically significant signal (n = 194, ROR = 2.49, 95% CI: 2.16–2.87, P < 0.001). No positive signals were found for the remaining drugs.

### 3.4 Analysis of signals associated with intervertebral disc herniation across different subgroups

We further conducted subgroup analyses by gender and age for the three drugs with positive signals: teriflunomide, natalizumab and ocrelizumab (Table 2). Results from the gender subgroup analysis indicate that the number of female participants (n = 585) was consistently higher than that of male participants (n = 153). However, when focusing on pharmacovigilance signals, the signal strength was slightly higher in the male subgroup than in the female subgroup, a trend that was particularly pronounced in the analysis of ocrelizumab. Specifically, in the gender-subgroup signal analysis for ocrelizumab, the male subgroup exhibited a positive signal (n = 43, ROR = 2.74, 95% CI: 2.03–3.70, P < 0.001), whereas this positive signal was not observed in the female subgroup. Furthermore, in the analysis of age subgroups, all three drugs exhibited positive pharmacovigilance signals in the 18–45 (n = 185) age group, negative signals in the 0–18 age group (n = 1), and varying signal strengths in other subgroups. It is worth noting that this phenomenon was more pronounced in the analysis results for ocrelizumab; in this population, only the 18–45 age group (n = 66, ROR = 3.46, 95% CI: 2.71–4.41, P < 0.001) exhibited a positive signal that met the threshold set for this study.

**TABLE 2:** Subgroup analysis of three drugs with positive warning signals based on age and gender.

Drugs	Subgroup	N	ROR (95% Cl)	PRR (χ^2^)	EBGM (EBGM05)	IC (IC025)	Bonferron_P_value
Teriflunomide	Total	236	**3.05(2.68–3.47)**	**3.05(321.90)**	**3.03(2.67)**	**1.60(1.40)**	**6.17721720914971e-68**
​	Male	44	**3.83(2.85–5.16)**	**3.83(91.60)**	**3.82(2.84)**	**1.93(1.41)**	**8.02924696364239e-18**
​	Female	172	**2.51(2.16–2.91)**	**2.51(154.22)**	**2.49(2.14)**	**1.32(1.08)**	**1.53653350130318e-31**
​	0–18	0	​	​	​	​	​
​	18–45	32	**2.45(1.73–3.47)**	**2.45(27.18)**	**2.44(1.72)**	**1.28(0.72)**	**0.000760481010836336**
​	45–65	142	**2.54(2.15–2.99)**	**2.53(130.13)**	**2.51(2.13)**	**1.33(1.07)**	**2.44194841117144e-26**
​	65–120	25	**3.93(2.65–5.83)**	**3.93(54.30)**	**3.91(2.64)**	**1.97(1.25)**	**1.07967358266414e-09**
Natalizumab	Total	332	**3.57(3.20–3.98)**	**3.57(605.96)**	**3.54(3.17)**	**1.82(1.65)**	**1.68917061246639e-129**
​	Male	66	**3.85(3.02–4.91)**	**3.85(138.01)**	**3.82(3.00)**	**1.94(1.52)**	**8.58502414810773e-28**
​	Female	266	**3.19(2.82–3.60)**	**3.18(392.89)**	**3.15(2.79)**	**1.66(1.47)**	**2.83262789942697e-83**
​	0–18	0	​	​	​	​	​
​	18–45	87	**4.18(3.38–5.17)**	**4.18(205.45)**	**4.10(3.32)**	**2.04(1.68)**	**2.10565050467633e-42**
​	45–65	105	**2.78(2.29–3.36)**	**2.77(117.90)**	**2.76(2.27)**	**1.46(1.16)**	**1.50662777028843e-23**
​	65–120	11	**4.05(2.24–7.33)**	**4.05(25.23)**	**4.04(2.24)**	**2.02(0.85)**	0.161974770693266
Ocrelizumab	Total	194	**2.49(2.16–2.87)**	**2.49(171.25)**	**2.48(2.15)**	**1.31(1.09)**	**4.01466590913183e-35**
​	Male	43	**2.74(2.03–3.70)**	**2.74(47.33)**	**2.73(2.02)**	**1.45(0.96)**	**3.92289842871758e-08**
​	Female	147	**2.28(1.93–2.68)**	**2.27(104.17)**	2.26 (1.92)	**1.18(0.93)**	**1.48746007760297e-20**
​	0–18	1	69.14 (9.60–497.86)	69.02 (66.19)	**68.16(9.47)**	6.09 (-1.09)	>0.999
​	18–45	66	**3.46(2.71–4.41)**	**3.45(113.10)**	**3.41(2.67)**	**1.77(1.36)**	**2.08916594097717e-22**
​	45–65	82	**2.08(1.67–2.59)**	**2.08(45.58)**	2.07 (1.67)	**1.05(0.71)**	**8.23444972787268e-08**
​	65–120	13	**3.2(1.85–5.51)**	**3.19(19.54)**	3.19 (1.85)	**1.67(0.69)**	0.486142769425224

Abbreviations: ROR, reporting odds ratio; PRR, proportional reporting ratio; IC, Information Component; EBGM, Empirical Bayesian Geometric Mean and P value, Adjusted P value. Bold values represent indicators that meet the predefined criteria for positive signals.

### 3.5 Analysis of the characteristics of adverse events in intervertebral disc herniation

**FIGURE 4:**
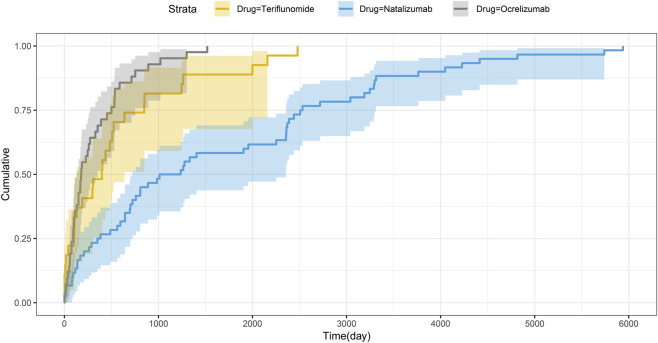
Cumulative distribution function of 3 drugs with positive pharmacovigilance signals by time-to-onset.

Among all 1,629 adverse event reports of intervertebral disc herniation, only 129 contained valid onset time data. Accordingly, all analyses regarding time to onset are presented as exploratory findings. Of the 129 eligible cases with documented onset time, 57 (44.19%) experienced the adverse event within 1 year after drug administration. We further summarized the median time to onset along with interquartile range (IQR), as well as the number of records with missing or invalid onset time for each individual agent. For teriflunomide, complete onset time information was available for 27 out of 236 relevant reports, with a median onset time of 399.0 days (IQR: 104.0, 743.5 days). For natalizumab, valid onset time data were obtained from 60 of 332 total reports, yielding a median onset time of 1,124 days (IQR: 376, 2,512 days). With respect to ocrelizumab, 42 out of 194 reports had documented onset time, with a median value of 179 days (IQR: 94, 488.5 days). Furthermore, teriflunomide exhibited a pattern of early failure, with adverse events concentrated in the early stages of treatment and a decreasing signal trend as exposure time increased (Table 3; Figure 4). In contrast, natalizumab and ocrelizumab exhibited a random failure pattern, with a more dispersed distribution of event occurrence times and no clear tendency toward temporal clustering.

**TABLE 3:** Weibull parameter test for intervertebral disc herniation-related adverse events associated with three drugs with positive warning signals.

Drugs	Shape parameter (95% CI)	Scale parameter (95% CI)	Type
Teriflunomide	0.67 (0.46–0.88)	456.63 (188.52–724.73)	Early failure
Natalizumab	0.85 (0.67–1.03)	1,552.76 (1,071.95–2033.58)	Random failure
Ocrelizumab	0.93 (0.71–1.15)	315.15 (207.55–422.76)	Random failure

CI, confidence interval; 95% CI, two-sided for Shape parameter and Scale parameter.

## 4 Discussion

The results of this study indicate that, among 13 immunosuppressive disease-modifying therapies, teriflunomide and natalizumab exhibited strong positive warning signals associated with intervertebral disc herniation, while ocrelizumab showed a relatively weaker positive signal compared to the former; the remaining 10 drugs showed no positive signals. Notably, these detected statistical discrepancies merely reflect disproportionality reporting trends in spontaneous pharmacovigilance data and cannot confirm definite causal or mechanistic relationships. All subsequent mechanistic interpretations presented below are speculative hypotheses only. We speculate that signal heterogeneity may stem from divergent pharmacological properties of DMTs. These drugs differ in immune targets, neuroprotective effects, regulation of spinal mechanical load and disc extracellular matrix metabolism, all of which may indirectly relate to intervertebral disc herniation.

Teriflunomide is a selective and reversible inhibitor of dihydro-orotate dehydrogenase, a key enzyme in the *de novo* pyrimidine synthesis pathway. It exerts both antiproliferative and anti-inflammatory biological effects, and this immunomodulatory action is primarily confined to the peripheral immune system (Gold and Wolinsky, 2011; Lycke et al., 2023). Although this drug can systemically regulate peripheral immune homeostasis, its low penetration efficiency across the blood-brain and blood-spinal fluid barriers makes it difficult to act on the central nervous system and the local innate immune microenvironment of the spine, thereby creating a potential risk for local immune imbalance and the development of degenerative disc disease. At the same time, teriflunomide fails to effectively inhibit the activity of key matrix-degrading enzymes, such as matrix metalloproteinases, in intervertebral disc tissue. This may disrupt the dynamic equilibrium between synthesis and degradation of the intervertebral disc extracellular matrix, ultimately leading to a reduction in the tensile strength of the annulus fibrosus collagen. This serves as a key molecular basis for the induction of intervertebral disc pathology and herniation (Seda et al., 2019). In terms of drug safety, teriflunomide demonstrates a favorable overall tolerability. Published studies have not reported notable adverse reactions such as muscle weakness or motor function impairment. It allows patients with multiple sclerosis to maintain near-normal limb muscle strength and activities of daily living (Gold and Wolinsky, 2011; Jozan et al., 2025). We hypothesize that preserved motor function may lead to prolonged physiological spinal loading, which could theoretically accelerate disc degeneration. However, this mechanical speculation cannot be confirmed in the current study. This may further lead to the spine bearing prolonged physiological mechanical loads, thereby accelerating the progression of intervertebral disc herniation. Furthermore, although teriflunomide participates in regulating pathways related to neural crest stem cells and skeletal homeostasis, it does not provide significant protection to vertebral endplate cells and cannot halt the progression of degenerative damage to the endplates. Potential endplate degeneration-related nutritional and metabolic impairment is proposed as a hypothetical pathway that may further promote the pathological progression of intervertebral disc herniation (Seda et al., 2019). In summary, teriflunomide may contribute to the development and progression of intervertebral disc herniation via multiple pathways. These include restricted peripheral immunomodulation, disrupted metabolism of the intervertebral disc matrix, sustained mechanical load on the spine, and a lack of protective effects on cartilaginous endplates. Concurrently, it also increases the detection rate of this condition to some extent in clinical practice.

As a monoclonal antibody targeting integrins, natalizumab can specifically block the infiltration of immune cells into the central nervous system and inhibit immune-mediated demyelination in multiple sclerosis; however, its pharmacological properties also carry a potential risk of inducing intervertebral disc herniation (De Keersmaecker et al., 2025). Similar to teriflunomide, natalizumab exhibits limitations in its immunomodulatory effects. The drug acts exclusively on central immune regions, significantly altering the distribution of B and T lymphocytes and suppressing central immune responses, but it cannot regulate the inflammatory homeostasis of peripheral connective tissues in the spine (Warnke et al., 2015). This immunological targeting bias may lead to persistent mediation of matrix degradation by pro-inflammatory factors such as IL-1β and TNF-α within the intervertebral disc, resulting in progressive damage to the annulus fibrosus structure. Pathologically, this exacerbates disc degeneration and ultimately contributes to the formation of intervertebral disc herniation. Additionally, recent studies have shown that natalizumab promotes myelin regeneration in acute demyelinating lesions and restores neural conduction, effectively rehabilitating motor and sensory functions in patients with multiple sclerosis (Fox et al., 2011). It is speculated that improved limb mobility may alter spinal mechanical loading patterns and potentially contribute to the observed statistical association.

Ocrelizumab is a CD20-targeted monoclonal antibody whose primary pharmacological mechanism involves B-cell depletion; it is widely used in the clinical treatment of multiple sclerosis (Margoni et al., 2022). This drug may be associated with warning signs of intervertebral disc herniation, and its pathogenic mechanisms can be analyzed by considering its immunomodulatory properties, adverse drug reactions, and metabolic changes. First, although ocrelizumab can deplete B cells and reduce peripheral inflammatory cytokine levels, the drug lacks a central neuroprotective effect. It cannot block the intrinsic degenerative pathological changes of the intervertebral disc and cannot fundamentally delay degenerative processes such as annular aging and nucleus pulposus dehydration (Hartung et al., 2023; Kornilov et al., 2024). Second, ocrelizumab may induce serum sickness-like adverse reactions in clinical settings, causing symptoms such as generalized weakness and joint pain (Rjeily et al., 2023). Prolonged physical discomfort may lead to abnormal activity patterns and an imbalance in the loading of spinal muscle groups, thereby exacerbating abnormal mechanical loads on the intervertebral discs. Furthermore, this drug may trigger metabolic and lipid pathway remodeling, altering the expression levels of bioactive lipids in peripheral tissues, which is hypothetically linked to disrupted disc microenvironment homeostasis (Kornilov et al., 2024).

Among anti-CD20 monoclonal antibodies, ocrelizumab showed a positive statistical association with the occurrence of intervertebral disc herniation, whereas ofatumumab and rituximab did not appear to promote intervertebral disc herniation. Notably, the following inter-drug mechanistic comparison is speculative. This difference may be explained by factors such as the drugs’ mechanisms of action, target binding characteristics, and the intensity of complement activation. Although ocrelizumab, ofatumumab, and rituximab are all CD20-targeting monoclonal antibodies, they exhibit significant differences in CD20 epitope binding sites, complement-dependent cytotoxicity (CDC) activity, the proportion of antibody-dependent cellular cytotoxicity (ADCC), and *in vivo* immunomodulatory effects (de Sèze et al., 2023; Carlson et al., 2024). Among these, ocrelizumab primarily acts via ADCC, with weaker CDC activation capacity compared to ofatumumab and rituximab (de Sèze et al., 2023; Førde et al., 2023). And ocrelizumab exhibits a more unique regulatory pattern on the peripheral and central immune microenvironments, as well as distinct effects on the inflammatory cytokine release profile and local tissue immune homeostasis (Carlson et al., 2024). Concurrently, ocrelizumab demonstrates broader and more sustained B-cell depletion effects and a distinct profile of immune-related adverse reactions in clinical practice. This differentiated immunomodulatory action may indirectly influence the local disc microenvironment and inflammatory repair processes, ultimately manifesting as a potential positive association with intervertebral disc herniation (Caldito et al., 2021; Roos et al., 2023); In contrast, the CDC-dominant effects of ofatumumab and rituximab, combined with subcutaneous administration (ofatumumab) or the characteristics of traditional chimeric antibodies (rituximab), result in a weaker impact on local pathological processes within the intervertebral disc. This may also lead to the identification of negative pharmacovigilance signals (de Sèze et al., 2023; Carlson et al., 2024).

Among the seven major classes of immunomodulatory drugs used in the treatment of multiple sclerosis, only integrin inhibitors, pyrimidine synthesis inhibitors, and certain anti-CD20 monoclonal antibodies exhibited positive pharmacovigilance signals for intervertebral disc herniation, whereas anti-CD52 monoclonal antibodies, fumaric acid derivatives, S1P receptor modulators, and immunosuppressive cytotoxic drugs did not exhibit such signals. As a speculative interpretation, this difference may be related to the target sites, tissue specificity, immune regulatory mechanisms, and clinical application characteristics of these four drug classes. Anti-CD52 monoclonal antibodies target only the CD52 antigen on the surface of immune cells to achieve deep depletion of peripheral lymphocytes. Their action is limited to the immune system and has no direct pharmacological effect on connective tissues such as the intervertebral disc nucleus pulposus and cartilage endplates. Furthermore, their core effect is immune reconstitution rather than interference with intervertebral disc matrix metabolism, which may explain the negative pharmacovigilance signals observed in disc injuries (Palmer, 2014; Dargahi et al., 2017). Furthermore, fumaric acid derivatives primarily exert their effects by activating the Nrf2 antioxidant pathway and inhibiting central NF-κB-mediated neuroinflammation. Their peripheral immunosuppressive effects are mild and highly targeted toward central glial cells; this process also does not disrupt the structural and metabolic balance of intervertebral disc tissue (Palmer, 2014; De Kleijn and Martens, 2020). In contrast, S1P receptor modulators exhibit strong blood-brain barrier permeability and primarily act on central S1P receptors to regulate the anti-inflammatory phenotype of microglia and astrocytes. Peripherally, they only mediate lymphocyte retention in lymph nodes; their immunosuppressive effects are limited and they exhibit no toxicity to intervertebral disc tissue, which may result in minimal impact on disc structural stability (Tavazzi et al., 2014; De Kleijn and Martens, 2020). Finally, although immunosuppressive cytotoxic drugs are broad-spectrum inhibitors, due to serious risks such as cardiotoxicity and secondary leukemia, clinical practice imposes strict cumulative dose limits and short treatment durations. Consequently, the population exposed to these drugs is small, significantly reducing the likelihood of generating positive warning signals for intervertebral disc herniation (Tavazzi et al., 2014; Dargahi et al., 2017).

We performed gender-stratified signal analysis for teriflunomide, natalizumab and ocrelizumab, all of which yielded positive pharmacovigilance signals for intervertebral disc herniation. The total number of adverse event reports was markedly higher in female patients. However, the signal strength was slightly greater among males. This gender heterogeneity may be jointly mediated by the epidemiological characteristics of multiple sclerosis, gender-specific biological differences in intervertebral disc herniation, and the gender-specific nature of drug effects. Specifically, multiple sclerosis itself exhibits a marked female susceptibility; the higher prevalence of female patients and the larger population exposed to the target drugs compared to males constitute the epidemiological basis for the higher number of adverse event reports in women (McGinley et al., 2021). Furthermore, the male population exhibits inherent characteristics regarding spinal biomechanical loads, the intensity of daily physical activity, and the structural composition of the annulus fibrosus collagen in intervertebral discs, which may result in higher susceptibility to degenerative disc disease and a faster rate of pathological progression (Machino et al., 2022). At the same time, the pharmacological effects of drugs such as ocrelizumab exhibit significant gender-specificity, with men being more prone to poor treatment response. This suggests that this population often requires longer treatment cycles or intensified treatment regimens, a characteristic that further increases the analysis signal of intervertebral disc herniation (Zaccone et al., 2025).

Subgroup analysis by age in this study revealed that adverse events related to teriflunomide, natalizumab, and ocrelizumab-specifically intervertebral disc herniation-exhibited a consistent positive pharmacovigilance signal only in the 18–45-year-old patient subgroup. This disparity may stem from the epidemiological characteristics of multiple sclerosis, the pathophysiological patterns of degenerative disc disease, and age-related heterogeneity in drug pharmacological effects. The core population affected by and treated for multiple sclerosis is concentrated in young-to-middle-aged adults. This age group constitutes the largest exposure cohort for the three disease-modifying therapies mentioned above, providing a critical demographic foundation for the consistent detection of positive signals (Oh et al., 2018). From the perspective of the pathophysiological progression of intervertebral discs, the discs in this population are still in the initiation and early progression stages of degeneration, with the water content of the nucleus pulposus, the elasticity of the annulus fibrosus, and structural integrity still maintained at relatively high levels. Under the combined effects of drug-mediated neural function repair and significant improvements in limb mobility, the dynamic biomechanical load on the spine increases substantially, potentially becoming a core mechanical driver triggering annulus fibrosus injury and nucleus pulposus herniation. Additionally, clinical symptoms in this age group are typically distinct, and symptoms related to intervertebral disc herniation are unlikely to overlap with those of neurological deficits caused by multiple sclerosis. This further enhances the detection efficiency of positive signals, ultimately resulting in consistent and stable positive pharmacovigilance signals for all three study drugs exclusively within the young-to-middle-aged subgroup.

It is worth noting that all analyses related to the time to adverse event onset are presented as exploratory findings, given the relatively small proportion of reports with valid onset time data relative to the total number of intervertebral disc herniation cases. Teriflunomide-associated intervertebral disc herniation exhibits early-onset signal characteristics, with adverse events concentrated in the initial phase of treatment; the signal gradually decreases as drug exposure time increases. In contrast, the timing of events associated with natalizumab and ocrelizumab is randomly distributed, with no clear tendency toward temporal clustering. Based on these temporal risk distribution patterns, clinicians should establish a time- and stratification-based, individualized prevention system for intervertebral disc herniation, formulating differentiated intervention strategies tailored to the temporal signal characteristics of these three drug classes. Specifically, for patients treated with teriflunomide, the first year following drug initiation should be designated as the core prevention window. A baseline spinal assessment should be completed prior to treatment initiation, and early intervention should include core muscle strength training, posture correction, and education on weight-bearing restrictions. For patients treated with natalizumab and ocrelizumab, given the random nature of risk occurrence, continuous prevention throughout the entire treatment course is required. Spinal health management should be incorporated into routine long-term follow-up, with patients receiving ongoing guidance to dynamically adjust activity intensity based on the status of neurological recovery. Furthermore, all patients with multiple sclerosis receiving treatment with the aforementioned three drugs should undergo systematic spinal health education prior to initiating therapy to enhance their awareness of the risk of intervertebral disc herniation, thereby improving the comprehensive safety management system for the entire course of modified disease-modifying therapy in multiple sclerosis.

This study conducted a real-world pharmacovigilance analysis based on the FAERS to systematically investigate the association between 13 immunosuppressive DMTs for multiple sclerosis and intervertebral disc herniation. Drawing on a large-scale dataset of spontaneous reports covering the mainstream immunosuppressive MS-DMTs, the study performed stratified analyses across multiple dimensions, including drug class, individual drugs, gender and age subgroups, and time to onset of adverse events. For the first time, this study systematically reveals the differences in risk signals and distribution characteristics between various MS-DMTs and intervertebral disc herniation, filling a gap in real-world pharmacovigilance evidence in this field and providing important reference data for clinicians to identify high-risk drugs and formulate stratified prevention and control strategies. However, given the nature of this spontaneous reporting database, this study has several limitations that require careful consideration in the interpretation of results and clinical application. First, the FAERS database, the data source for this study, is a spontaneous, voluntary reporting system. Inherent reporting biases are unavoidable. Additionally, missing demographic data may introduce potential bias to the results of this study. Furthermore, the database cannot accurately capture information on patients’ underlying spinal conditions or medical history, which may affect the accuracy and stability of drug disproportionality signals to some extent. Second, this study is a retrospective observational pharmacovigilance study that can only identify statistical associations between drugs and adverse events. It cannot establish a causal relationship between MS-DMTs and intervertebral disc herniation. The positive signals identified require further validation and confirmation through prospective cohort studies and large-scale clinical controlled trials. Furthermore, this study did not incorporate key exposure factors, such as drug dosage, into its stratified analysis. Therefore, prospective studies are still needed to further clarify the relationship between these factors and provide a higher level of evidence-based support for the safe and rational use of medications in clinical practice.

## 5 Conclusion

This FAERS-based analysis identified disproportionality reporting signals for teriflunomide, natalizumab, and ocrelizumab that warrant further pharmacoepidemiological and mechanistic investigation.

## Data Availability

The original contributions presented in the study are included in the article/Supplementary Material, further inquiries can be directed to the corresponding authors.
